# Children with Autism Spectrum Disorders Have “The Working Raw Material” for Time Perception

**DOI:** 10.1371/journal.pone.0049116

**Published:** 2012-11-21

**Authors:** Sandrine Gil, Patrick Chambres, Charlotte Hyvert, Muriel Fanget, Sylvie Droit-Volet

**Affiliations:** 1 Centre de Recherches sur la Cognition et l’Apprentissage, UMR CNRS 7295, University of Poitiers, Poitiers, France; 2 Laboratoire de psychologie Sociale et Cognitive, Blaise Pascal University, UMR CNRS 6024, Clermont-Ferrand, France; Duke University, United States of America

## Abstract

The aim of the present study was to investigate whether children with Autism Spectrum Disorders (ASD) have a deficit in time perception. Twelve ASD children of normal intelligence and twelve typically developing children (TD) - matched on sex, chronological age, and mental age – performed four temporal bisection tasks that were adapted to the population. Two short (0.5 to 1 s and 1.25 to 2.5 s) and two long duration ranges (3.12 to 6.25 s and 7.81 to 16.62 s) were thus examined. The findings suggested that the perception of time in bisection is not impaired in ASD.

## Introduction

Autism spectrum disorders (ASD) are a group of neurodevelopmental disorders whose etiology is as yet poorly understood [1], [2]. Nevertheless, ASD have a huge impact on three domains of competence that constitute the triad criteria used for the diagnosis of autism (DSM-IV-TR; American Psychiatric Association 2000; ICD 10; World Health Organization 1992). First, individuals with ASD show a marked impairment in social interaction, leading to a restricted and impaired ability to share common experiences with others. Second, their modes of communication, both verbal and non-verbal, are impaired. Language development is retarded and/or language is used inappropriately in pragmatic contexts [3]. The decoding of important communicative signals such as emotional facial expressions or prosody is also disrupted [4]–[6]. Third, individuals with ASD present stereotyped and repetitive behaviors, and restricted centers of interest.

Clinical reports suggest that subjects with autism have another major deficit - a timing deficit [7]–[9]. Therefore, the ability to time events and actions is crucial for the adaptation of behavior to the physical and social environment [10]–[13]. Indeed, the ability to process time correctly ensures that social interaction with others progresses harmoniously and permits, for example, intersubjectivity at an early stage of development [14], [15]. It allows children to understand and learn the dynamic of language (i.e. turn-taking) and non-verbal social cues [16]. It also makes it possible to predict the timing of events. In this domain, some researchers have suggested that autistic children often produce repetitive behaviors to compensate for their difficulties in predicting incoming events [17]. Taken together, the diagnostic criteria and the clinical reports have therefore prompted speculation that difficulties in processing time might be an important factor in autistic disorders and might explain a part or even all of the primary symptoms [17].

Over the last decade, a relative small number of studies has examined the ability to process time information in ASD individuals with classical timing tasks, for a review see [18]. However, the results of these studies are inconsistent and in some cases contradictory. Using a temporal reproduction paradigm, Szelag et al. [25] showed that children with autism were totally unable to reproduce visual and auditory durations, and concluded that such children have a major deficit in duration judgment. Although neither Martin et al. [23] nor Gowen and Miall [21] found that ASD children had major difficulties in processing time, they did observe that these children were less accurate than control subjects in a time reproduction task and a temporal discrimination task (i.e. temporal generalization task) respectively. In the same way, Maister and Plaisted-Grand [22] found children with ASD less accurate than controls in duration reproduction. Using a generalization task, Falter et al. [20] also found a reduced interval timing sensitivity in ASD participants. In a temporal bisection task, in which the participants had to categorize durations as more similar to a short or a long anchor duration, Allman et al. [19] showed that individuals with ASD tended to overestimate durations although no specific impairment of their time sensitivity was observed. In contrast, Wallace and Happé [26] obtained similar performance in individuals both with and without autistic disorders in three different temporal tasks: verbal time estimation, time production and time reproduction. These authors therefore concluded that time perception in children and adolescents with autism is intact. Similarly, Mostofsky et al. [24] showed that people with autism were as accurate as control subjects in their explicit judgments of temporal intervals. Therefore, faced with this lack of consistency in the results of studies of time perception in autistic individuals, the aim of our study was to add results in this research area by using a bisection task that has not been often used up until now except by Allman and collaborators [19], and by examining a wide range of durations from a few hundreds of milliseconds to several seconds.

According to the most popular model of time processing, elaborated by Gibbon, Church and Meck [27] in the framework of the Scalar Expectancy Theory (SET) [28], the efficient processing of time results from the interaction between three mechanisms which are involved at different levels of the information processing: a clock, memory processes and decision processes. The clock is thought to consist of a pacemaker that emits pulses which pass into an accumulator via a switch which closes when a duration has to be timed. The functioning of the pacemaker is known to be principally arousal-based [29]–[33], and that of the switch attention-based [34]. The internal clock system is thus considered to be the primary level at which the representation of time is formed, before higher-level cognitive processes – memory and decision – intervene. However, a growing body of literature suggests the processing of durations shorter than 1–2 s. does not involve the same mechanisms as that of longer durations. Short durations are thought to require automatic processes, whereas long durations involve controlled processes [35]–[38]. Because the processing of time requires the ability to maintain attention throughout the entire duration to be timed [39], the processing of long durations (>2 s.) are more demanding both in terms of working memory and attention than the processing of shorter ones. It’s interesting to note that previous studies in people with ASD have tested a wide range of durations, below or near a second [20], [21], [24], less than eight seconds for others [19], [22], [23], [25] and until forty-five seconds for another [26]. However, findings were discrepant whatever the durations used. In addition, the SET model of temporal processing involves what is called the scalar property, i.e., the fact that the variability of time estimates increases proportionally to the mean duration value to be timed. In others words, time performance variability constitutes a constant across a relative scale of durations [40], [41]. Only two studies have examined this mathematical propriety of time in ASD populations, but each leads to a different conclusion, although the two studies used, respectively, long and short durations. Using long durations (i.e. 2 to 8 s.) Allman et al. [19] found that the scalar property was not fully respected in a bisection task performance by the group of ASD people, whereas Falter et al. [20] found it preserved in a generalization task performance using shorter durations (i.e. 0.5 to 1.5 s).

Numerous studies have shown a correlation between the ability to process time and intellectual efficiency [42], [43], the individuals with low IQ having low sensitivity to time. In particular, ASD is often associated with mental retardation (DSM-IV-TR). Consequently, the difference in timing performance between ASD and control groups might be mainly due to differences in general cognitive functioning rather than to a deficit in temporal processing mechanisms *per se* [19], [24]. In support of this, Allman and collaborators [19] indicate in ASD children a significant correlation between an index of time sensitivity and subjects’ scores in tasks reflecting language and working memory capacities. Consequently, if the mental age of the children with autism is the same that their age control group children, we might suppose that differences in temporal performance would be reduced. In addition, the poor performance found in children with autism might be always obtained if they are insufficiently motivated to do a particular activity. Indeed, it is well known that ASD children have restricted fields of interest and are not motivated when the task that they are required to do lies outside of their field of interest [44]. Therefore, in our study we used high functioning children with autism, characterized by no mental retardation, compared with mental age-matched typically developing children. Moreover, in an attempt to maintain ASD children’s interest in the task and promote a high level of performance, we choose to use reinforcements, i.e. a gift, that play a critical role in motivation [44], [45].

The purpose of the present study was thus to test children with ASD using the temporal bisection task which has frequently been administered to children [46]–[48], and that is considered to be a “pure” perceptual task [49], that minimizes the intervention of higher cognitive processes, i.e. memory [46], [50], [51]. In the temporal bisection task, participants are initially presented with a short and a long anchor duration. They then perform a number of trials and have to judge whether the probe durations (equal to the anchor durations or representing intermediate values) are more similar to the short or the long anchor duration. Each participant performed two sets of bisection tasks: (1) a first set of two bisection tasks examined short duration ranges, i.e. 0.5/1 s. and 1.25/2.5 s, (2) another set of bisection tasks examined long durations ranges, i.e. 3.13/6.25 s and 7.81/16.63 s. As previously mentioned, we used these four duration ranges in order to investigate the scalar properties of time with short and the long durations in the ADS children compared to control children, but also to compare their performance with duration ranges that differ in the demands placed upon attention and working memory [52], [38]. Moreover, we slightly modified the bisection task to increase and maintain the children’s interest throughout the task [44], [45]. First, in order to increase motivation, each child was told that they would receive a gift at the end of the experiment. A photo of this gift was presented and gradually revealed during the bisection task, with the gift being given at the end of the session. This gift was chosen for each child as a function of their specific interests as reported by their parents. Second, tasks presented to children with autism have to be well structured in order to improve their understanding of what is expected of them and encourage their self-control [53], [54]. Consequently, in order to show the children how many trials they had completed in the temporal bisection task and how many remained, we presented a set of visual markers (empty circles) representing the trials and these were filled in one-by-one as the child completed the individual trials.

## Methods

### Participants

Seventeen ASD children were first recruited from various associations. Children and parents gave written informed consent to participation as required by the Clermont-Ferrand Sud-Est VI Statutory Ethics Committee (CPP), which approved the present study. Criteria for exclusion were mental retardation, absence of language, the subject attrition during all bisection tasks, and inability to perform the training phase of the proposed procedure correctly. The final group of children with autism spectrum disorder (ASD) comprised twelve normal intelligence male children, aged from 9 to 17 years. Nine were diagnosed with Asperger’s Syndrome (AS) and three as exhibiting High-functioning autism or non-specified Pervasive Developmental Disorder (PDD). All ASD subjects had a formal diagnosis made by experienced independent clinicians before the study and based on DSM-IV criteria. Twelve volunteer typically developing children (TD), aged from 8 to 16 years, were recruited for the control group and the group means were matched on sex, chronological age (CA), and mental age (Table 1). Table 1, for each group of participants, shows the different composite scores from the Wechsler Intelligence Scale for Children (WISC-IV): Verbal Comprehension index (VCI), Perceptual Reasoning Index (PRI), Working Memory Index (WMI), Processing Speed Index (PSI), Full Scale IQ (FSIQ). There were no significant difference between the groups on either chronological age (CA) (Mann-Whitney, *U*(65) = −.404; *p* = .71), nor any composite score, all p>.1.

**Table 1:** For each group of children (ASD: Autism Spectrum Disorders; TD: Typically Developing), means and standard deviations for each composite score from the Wechsler Intelligence Scale for Children (WISC-IV): Verbal Comprehension index (VCI), Perceptual Reasoning Index (PRI), Working Memory Index (WMI), Processing Speed Index (PSI), Full Scale IQ (FSIQ).

	ASD		TD	
	Mean	SD	Mean	SD
CA	13	2.49	13.21	2.32
VCI	109.25	27.25	101.63	21.47
PRI	100.87	18.80	102.27	17.01
WMI	91.37	19.47	100.81	10.57
PSI	83.25	25.03	101.45	22.12
FSIQ	94.37	22.39	101.45	19.49

### Material

The children were individually tested at home in a quiet room. Visual paper-based material was used to explain the various stages involved in the bisection task procedure. Consequently, screenshots were printed indicating twenty-one circles representing the different trials in a block. These were originally empty and were filled one-by-one as the trials progressed throughout the block. When a block of twenty-one circles had been filled (twenty-one trials completed), the document revealed a picture of a part of the personal gift that each child was to receive at the end of the session. For the bisection task, a PC was used to control the experimental events using E-Prime software (1.2, Psychology Software Tools, Pittsburg, PA). The temporal stimulus was a blue 7×3 cm rectangle presented in the center of the computer screen. The picture presenting the twenty-one circles appeared on the computer screen between each trial. In addition, in the training phase of the bisection task, pictures of red and green lights were used as feedback, with the former indicating incorrect and the latter correct discrimination of the probe durations.

### Procedure

Each child completed four bisection sessions, for each duration range respectively, with the session order being counterbalanced across subjects who performed two sessions a day to avoid fatigue. Two bisection tasks corresponded to short duration ranges (<2 s.). Range 1: the short and the long anchor durations were 0.50 and 1.0 s and the probe durations 0.50, 0.58, 0.67, 0.75, 0.83, 0.92 and 1.0 s; Range 2: the anchor durations were 1.25 and 2.5 s and the probe durations 1.25, 1.46, 1.67, 1.87, 2.083, 2.29 and 2.5 s. Two other bisection tasks corresponded to long duration ranges (>2 s.). Range 3: anchor durations of 3.13 and 6.25 s, and probe durations of 3.13, 3.65, 4.17, 4.69, 5.21, 5.73 and 6.25 s; Range 4 used 7.81 and 16.63 s as anchor durations, and 7.81, 9.12, 10.42, 11.73, 13.03, 14.33 and 16.63 s as probe durations. In each bisection session, the child had to estimate whether the presentation time of the stimulus was more similar to the previously presented short or long anchor stimulus duration. Responses (i.e. short or long) were given verbally, with the experimenter then pressing the corresponding response key, in order to reduce motor skills’ impact on performance. Each bisection task consisted of three phases. In the first phase, the short and the long anchor durations were presented three times each. Then, in a second phase, the participants were trained to respond “Short” or “Long” on eight trials, four for each anchor duration. In this training phase, feedback was given in the form of the green or red light depending on whether the provided response was correct or not. When the participants responded correctly on 75% of the trials, the training terminated and the third (test) phase started. This consisted of three blocks of twenty-one trials each (i.e. sixty-three trials in total), with the seven probe stimulus durations being presented three times in each block. At the beginning of each block, the anchor durations were again presented three times each. Between each trial, the picture of the twenty-one circles with one more filled appeared on screen. In addition, between each block, a picture of a part of the present was revealed to the child using the visual printed material.

## Results

As mentioned in introduction, because shorter and longer duration ranges involve different kind of processes [35]–[39], and in order to clarify this section, results are presented first for the two short duration ranges, and second for the two long ones.

### Short Duration Ranges (Range 1: 0.50/1.0 s – Range 2: 1.25/2.5 s)

**Figure 1:**
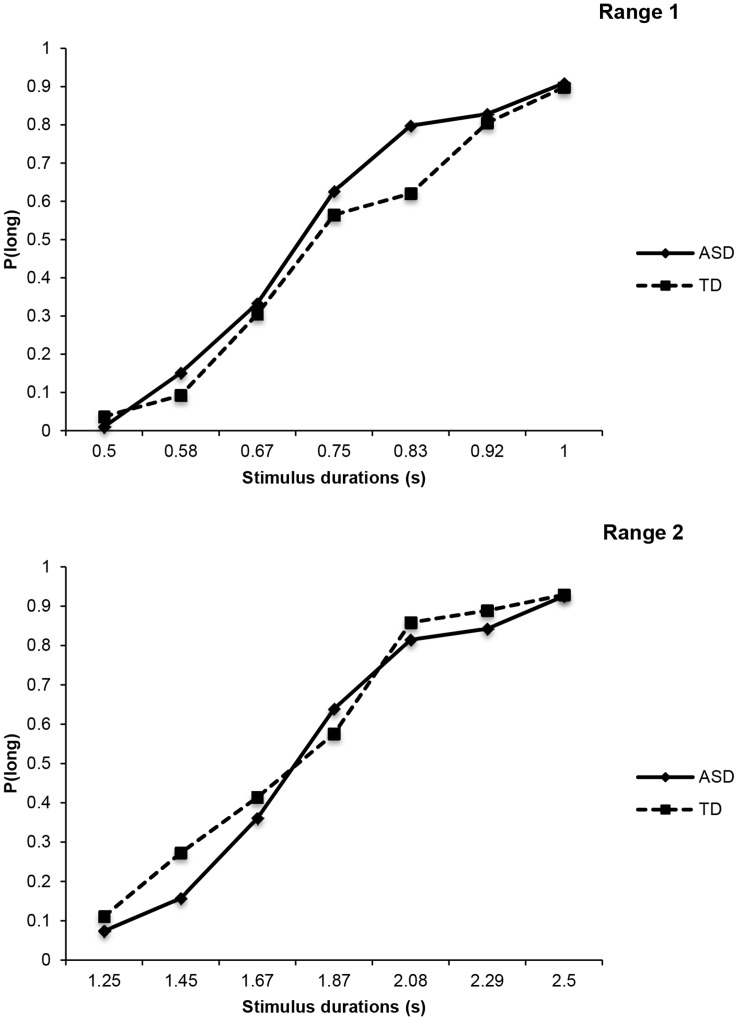
Mean proportion of “long” responses plotted against stimulus durations, for both ASD and TD participants, and for duration range 1 (upper panel), and duration range 2 (lower panel).

An overall ANOVA, with two within-subjects factors (2 durations ranges and 7 stimulus durations per range), and group (ASD *vs.* TD) as a between-subjects factor, was run on the proportion of long responses (see Figure 1). This analysis yielded neither a significant main effect of group, *F*(1, 22) = .11, *p* = .74, nor any interaction involving this factor, all *ps*>.1. There was only a significant main effect of stimulus duration, *F*(2.57, 56.59) = 160.45, *p*<.001, and a significant main effect of duration range, *F*(1, 22) = 5.87, *p* = .02. This finding indicates that the proportion of long responses increased with stimulus duration irrespective of group (with or without autism). This suggests that children with ASD seem to be able to discriminate probe durations in the same way as other children.

To evaluate temporal discrimination in ASD children in greater detail, we calculated three supplementary indexes: BP, DL and WR (Table 2). These three indices were derived from the intercept and the slope of the logarithmic regression that significantly fitted the individual bisection functions for each subject. The Bisection Point (BP) corresponds to the duration value at which short and long responses occur with equal frequency (*p*(long) = .50). Mann-Whitney two by two comparisons showed that the BP values did not differ between groups for either ranges (Range 1: *U*(51) = −92, *p* = .38; Range 2: *U*(54) = −.74, *p* = .49). The Difference Limen (DL) is equal to half the difference between the stimulus duration giving rise to 75% of “long” responses and the duration giving rise to 25% of such responses. The DL is thus an index of absolute time sensitivity and indicates the shortest discriminable difference between the employed stimulus durations. We found no difference in DL values between the groups of subjects for either of the two ranges examined (Range 1: *U*(47) = −1.17, *p* = .26: Range 2: *U*(51) = −.92, *p* = .38). In sum, the minimum observable temporal difference was similar in the children with and without autism.

**Table 2:** Mean bisection point (BP), difference limen (DL), and Weber ratio (WR) for the short (0.5/1.0-s) and the longer (1.25/2.5-s) duration ranges in children with autistic spectrum disorder (ASD) and typically developing children (TD).

Group	Durations range 1 (0.5 to 1 s)						Durations range 2 (1.25 to 2.5 s)					
	BP		DL		WR		BP		DL		WR	
	Mean	SD	Mean	SD	Mean	SD	Mean	SD	Mean	SD	Mean	SD
ASD	728.6	67.74	140.3	50.97	.19	.05	1781	181.5	378.7	182.1	.21	.09
TD	778.5	113.0	153.9	50.51	.19	.04	1696	195.9	343.3	62.22	.21	.05

**Figure 2:**
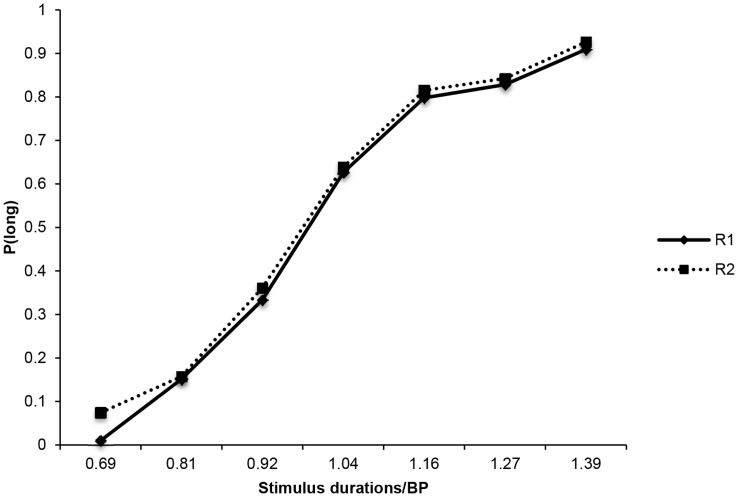
Superimposition of the psychophysical functions for duration range 1 (R1) and duration range 2 (R2) in ASD participants.

The Weber Ratio (WR) corresponds to the DL divided by the BP. The WR is also a parameter of time sensitivity. A lower Weber Ratio value indicates a better sensitivity to time [48]. However, compared to the DL, it provides an index not of absolute but of relative sensitivity to time. Moreover, it allows us to test the scalar property of variance, which is a sort of Weber’s Law [28]. According to this property, the variability in perceived time increases proportionally to the mean duration value to be timed. Consequently, the WR, which is considered to be a type of coefficient of variation (DL/BP), should remain constant across duration ranges if the scalar property holds [40], [55]. An ANOVA was therefore run on Weber Ratio values with duration range as a within-subjects factor, and group as a between-subjects factor. This statistical analysis revealed no main effect of duration range, *F*(1, 20) = .44, *p* = .51, nor of group, *F*(1, 20) = .1, *p* = .76, and no interaction involving these two factors, *F*(1, 20) = .000, *p* = .99. Finally, to test the scalar property in ASD time performance in addition to the statistical examination of Weber Ratio values, we tested the superimposition of the psychophysical functions for the different duration ranges. The proportion of “long” responses for the two duration ranges was therefore plotted against a relative duration scale by dividing each stimulus duration by the corresponding BP for the duration range used [50]. Figure 2 shows the psychophysical functions obtained with this method, and indicates that the ASD children’s functions for the two duration ranges superimposed consistently well, consistent with the scalar properties of variance.

### Long Duration Ranges (Range 3: 3.13/6.25 s – Range 4: 7.81/16.63 s)

**Figure 3:**
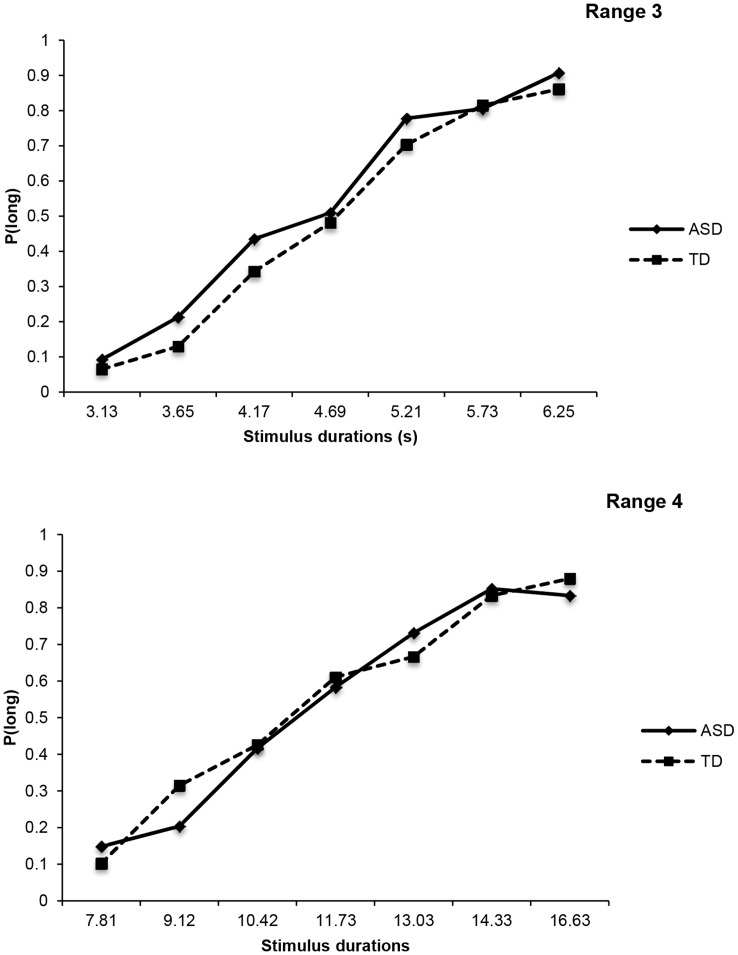
Mean proportion of “long” responses plotted against stimulus durations, for both ASD and TD participants, and for duration range 3 (upper panel), and duration range 4 (lower panel).

A 2×7×2 ANOVA was conducted on the proportion of long responses. The main effect of stimulus duration was significant, *F*(3.47, 72.42) = 142.59, *p*<.001, and indicated that the proportion of long responses increased with the stimulus duration values. Neither the between-group effect, *F*(1, 22) = .21, *p* = .65, nor duration range effect, *F*(1, 22) = .79, *p* = .38, nor any interaction involving these factors were significant (all *ps*>.1). As Figure 3 illustrates, these results suggest that ASD and TD children performed equally well when asked to classify the different stimulus durations in a bisection task, even when long durations were used.

**Figure 4:**
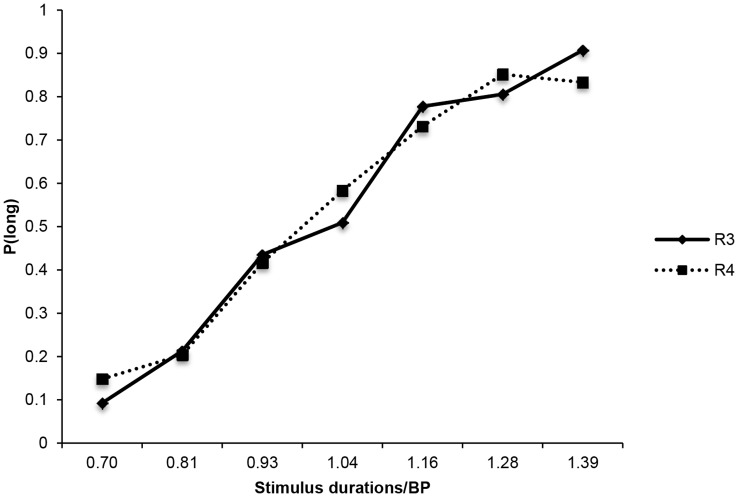
Superimposition of the psychophysical functions for duration range 3 (R3) and duration range 4 (R4) in ASD participants.

As for short duration ranges, analyses were conducted on the BP, DL and WR values calculated as previously described (see values in Table 3). Planned comparisons demonstrated that the performance of the ASD and TD groups did not differ significantly for the 3.13/6.25 s duration range when the BP was used (*U*(59) = −.75, *p* = .48) nor when the DL was analysed (*U*(71) = .95, *p* = .98), and the same picture was obtained with the 7.81/16.63-s duration (BP: *U*(66) = 0, *p* = 1; and DL: *U*(57) = −.55, *p* = .61). These findings confirmed that the two groups had similar temporal discrimination abilities. Moreover, relative sensitivity to time was examined in the same way as for short ranges using a 2×2 ANOVA run on the WR values. This revealed no significant effect of duration range, *F*(1, 21) = 2.77, *p* = .11, group, *F*(1, 21) = .05, *p* = .82, or two-way interaction, *F*(1, 21) = .12, *p* = .73. In addition, the psychophysical functions in the two duration ranges (see Figure 4) for ASD participants superimposed well, thus confirming that their time performance exhibited the scalar variability property. In sum, analyses revealed that ASD children’s time perception performance exhibits similar properties, i.e. discriminability and sensitivity, as typically developing children, even for durations longer than three seconds.

**Table 3:** Mean bisection point values (BP), difference limen (DL), and Weber ratio values (WR) for each duration range (range 3 vs. range 4) and group participants (ASD vs. TD).

Group	Durations range 3 (3.12 to 6.25 s)						Durations range 4 (7.81 to 15.62 s)					
	BP		DL		WR		BP		DL		WR	
	Mean	SD	Mean	SD	Mean	SD	Mean	SD	Mean	SD	Mean	SD
ASD	4459	562.5	997.3	368.1	.22	.07	11300	1791	2724	1534	.23	.10
TD	4644	473.0	950.8	211.3	.20	.04	11011	1639	2563	664.5	.23	.05

### Short and Long Duration Ranges Comparison

Finally, because the same subjects took part in all sessions, we examined whether sensitivity to time was constant across the short and long duration ranges by performing an ANOVA on mean WR values for all four duration ranges. This analysis revealed neither a main effect of duration range, *F*(2.26, 43.1) = 2.46, *p* = .09, nor of group, *F*(1, 19) = 1.62, *p* = .22, nor any interaction between the two factors, *F*(3, 57) = .56, *p* = .64. These findings show that the time perception performance of both groups of children exhibited the scalar property across both the examined duration ranges, short and long.

## Discussion

Clinical reports have suggested that individuals with autism spectrum disorder have difficulties in conceptualizing the passage of time and in controlling the time taken to achieve an objective [7], [56], [57]. Despite this, the findings reported by experimental research have been contradictory, with some studies concluding that ASD subjects exhibit a deficit in time perception or atypical temporal behavior [19]–[23], [25], whereas other results have failed to indicate any time atypicalities [24], [26]. Using a bisection task, our results demonstrated that the children with autism spectrum disorder but without mental retardation had a high ability to discriminate durations and that they exhibited no more variability in their time judgements than control children for both the short (<2 s) and long (from 2 to 17 s) duration ranges. In addition, their time discrimination exhibited the fundamental scalar timing properties found in both humans and animals [58], [28]. ASD children’s sensitivity to time (WR) was constant across different duration values that cover a large range of durations (i.e. 0.5 to 16.63 s), consistent with Weber’s law. In sum, our study, which used a temporal bisection task, provided evidence indicating that the perception of time in people with autism might be intact. Our conclusion is thus entirely consistent with conclusions previously presented by Mostofsky et al. [24] and Wallace and Happé [26]. Concerning the scalar property, our findings are consistent with the work of Falter et al. [20] who found that the Weber’s Law hold for the timing in ASD participants, but contradictory to Allman et al.’s results [19]. Nevertheless, as highlighted by the former, a main difference between these two studies is that, in Allman and colleagues’ work, ASD participants were low IQ. A substantial body of research has described a link between intellectual efficiency and the ability to process time [42], [43], [59]. Consequently, the present findings suggest that it is necessary to exercise caution about the temporal performance of pathological individuals, when low IQ may be a mediating factor in performance. The use of HFA and AS scores to define a population without mental retardation who are, moreover, matched on a mental efficiency with control participants, therefore seems to be a viable way to study autism *per se*. Specifically, our study suggested that, with this population without mental retardation, autism would not be associated with deficits in the perception of durations in the range of hundreds of milliseconds or seconds.

However, the question arises of how to explain the discrepancy of results between studies examining time perception in autism, beyond the general IQ problem. It is interesting to note that the time perception can be considered as task-dependent. Indeed, the different temporal tasks used in different studies with ASD participants (i.e. bisection, reproduction, production, generalization) call upon certain cognitive processes (e.g. memory, decision) at different stages of time processing [60], [61], [46], [51]. Within this framework, previous studies examining time perception in people with autism were heterogeneous in terms of the tasks used. This may be a source of discrepancy between the results of different studies. By way of conclusion, we can propose that the temporal deficits observed in autistic individuals in previous studies did not result from a specific deficit in the mechanism involved in producing the “raw material” for the processing of time, i.e. an internal clock mechanism, but rather from other cognitive functions that interfere with the final time judgment, and that were required to a greater or less extent as a function of the procedures used. According to this account, the impairments in time processing observed in previous studies might be attributable to limitations to cognitive processes (e.g., attention, memory, motor or decision-related abilities) the involvement of which we have tried to minimize in our study.

As far as attention is concerned, although many studies of ASD have examined attentional capacities of such individuals, no clear consensus has as yet been reached. However, it is interesting to note that attention-deficit hyperactivity disorder symptoms often seem to co-occur in ASD [62], [63]. In addition, a number of recent studies have revealed that variation in the attention capacities of children, as evaluated using neuropsychological tests, explains a large part of the variability in their time sensitivity as a function of age [64], [65], [59], [66]. Moreover, a significant body of literature has linked attention difficulties in autism to the complexity of the task employed or the motivation to perform it [67], [68]. By introducing certain methodological adaptations to the temporal bisection task, such as trial markers and a gift as reinforcement, it seems that we might have circumvented the problem of lack of motivation and have increased sustained attention throughout the task in ASD children, thus potentially eliminating the influence of their attention-related difficulties on their time judgments. However, the fact that this methodological adaptation was not really tested in our work (e.g. examine the effect on the performance of the procedure with and without adaptation within the same ASD participants) it is difficult to conclude to its benefit. Moreover, one limitation of our study is that the small number of participants did not permit us to examine potential individual differences in task performance. There is some previous evidence that ASD participants may show considerable heterogeneity of performance. For example, whereas the sensory modality (visual vs. auditory) of presented stimuli systematically impacts on temporal processing in typical individuals with a lower sensitivity to visual compared to auditory stimuli [69], findings show high-subject heterogeneity in ASD people’s performance as a function of the modality, sometimes involving better performance with visual stimuli than auditory, or sometimes worse [70], [71], [72]. Consequently, a possibility is that discrepant findings in previous works reflect the extreme heterogeneity in this population in terms of their sensitivity to time as a function of the stimuli used. In addition, because motor skills are also known to be impaired or retarded in ASD [73], in our study, we prevented the autistic children’s motor difficulties from interfering with timing by using a temporal bisection task that minimized the role of motor behavior, compared to a temporal reproduction task for example.

Finally, we can also relate our findings to one current mainstream hypothesis concerning autism, which suggests that it involves atypical information processes, where information seems to be primarily processed analytically – or locally – rather than in the typical holistic – or global – way [74]. This processing bias implies that people with autism do not naturally tend to integrate elements into a higher level of organization, as suggested by the Weak Central Coherence hypothesis [75], [76]. Although speculative at this point, it is thus conceivable that, whereas ASD individuals have the raw material to perceive time, their difficulties on some temporal judgements suggested in clinical reports reflect fundamentally atypical information processing involving higher cognitive processes. Indeed, time constitutes complex information that needs to be integrated without any particular sense organ being dedicated to time processing, and in variable contexts. Although not related to timing tasks per se, some findings related to the management of temporal elements are consistent with this interpretation. For example, findings showed that people with autism perform as the same way as a control group to plan a movement when instructions as to task requirements were given immediately prior to the movement, whereas they failed to perform as well when the instructions were only accessible in the movement environment [77]. In the same way, a recent study showed that on a perceptual simultaneity task, people with ASD showed impaired temporal integration, but over-performed in temporal resolution [71]. It is thus important to further investigate the specificity of time disturbances in ADS individuals as a function of type of task employed.

To conclude, autism spectrum disorder can be explained at different levels of information processing, which is why the investigation of the specific abilities of such individuals is so challenging, in particular considering timing abilities. Moreover, the assessment of timing abilities appears to be of particular interest when consider interaction deficit in autism, that is their problem in properly regulating social interactions, and the fundamental link between timing and both verbal and non-verbal communication [19], [20]. Within this perspective, our study therefore extends the literature by demonstrating that ASD children seem to have the raw material for the discrimination of durations. However, further work is necessary to clarify the specific contexts in which autistic children’s timing may be impaired and why.

## References

[1] Geschwind DH, Levitt P (2007) Autism spectrum disorders: developmental disconnection syndromes. Current Opinion in Neurobiology 17: 103–111. 17275283 10.1016/j.conb.2007.01.009

[2] Persico AM, Bourgeron T (2006) Searching for ways out of the autism maze: genetic, epigenetic and environmental cues. Trends in Neurosciences 29: 349–358. 16808981 10.1016/j.tins.2006.05.010

[3] Loukusa S, Moilanen I (2009) Pragmatic inference abilities in individuals with Asperger syndrome and high-functioning autism. A review. Research in Autism Spectrum Disorders 3: 890–904.

[4] Castelli F (2005) Understanding emotions from standardized facial expressions in autism and normal development. Autism 9: 428–449. 16155058 10.1177/1362361305056082

[5] De Gelder B, Vroomen J, van der Heide L (1991) Face recognition and lipreading in autism. European Journal of Cognitive Psychology 3: 69–86.

[6] McCann J, Peppé S (2003) Prosody in autism spectrum disorders: a critical review. International Journal of Language and Communication Disorders 38: 325–350. 14578051 10.1080/1368282031000154204

[7] Boucher J (2001) Lost in a sea of time: Time-parsing and autism. In: Hoerl C, McCormack T, editors. Time and memory. Oxford: Clarendon Press. 111–135.

[8] Peeters T, Gillberg C (1999) Autism, Medical and Educational Aspects, 2^nd^ edn. London: Whurr Publishers Ltd.

[9] Wing L (1996) The autistic spectrum. London: Jessica Kingsley Publishers.

[10] Bateson M (2003) Interval timing and optimal foraging. In: Meck WH, editor. Functional and neural mechanisms of interval timing. Boca Raton, FL: CRC Press. 113–141.

[11] Fraisse P (1977) Des différents modes d’adaptation au temps. In: Fraisse P et al.., editors. Du temps biologique au temps psychologique. PUF, Paris. 9–20.

[12] Ivry RB (1996) The representation of temporal information in perception and motor control. Current Opinion in Neurobiology 6: 851–857. 9000026 10.1016/s0959-4388(96)80037-7

[13] Meck WH, Benson AM (2002) Dissecting the brain’s internal clock: How frontal-striatal circuity keeps time and shifts attention. Brain and Cognition 48: 195–211. 11812042 10.1006/brcg.2001.1313

[14] Kasari C, Sigman M, Yirmiya N, Mundy P (1993) Affective development and communication in young children with autism, Enhancing children’s communication: Research foundations for intervention, Communication and language intervention series, Vol. 2 (pp. 201–222). Baltimore, MD, England: Paul H. Brookes Publishing, xix, 423 pp.

[15] Wimpory DC, Hobson RP, Williams JMG, Nash S (2000) Are infants with autism socially engaged? A study of recent retrospective parental reports. Journal of Autism and Developmental Disorders 30: 525–536. 11261465 10.1023/a:1005683209438

[16] Baron-Cohen S (1989) Social and pragmatic deficits in autism: Cognitive or affective? Journal of Autism and Developmental Disorders 18: 379–402. 10.1007/BF02212194 3049519

[17] Allman MJ (2011) Deficits in temporal processing associated with autistic disorder. Frontiers in Integrative Neuroscience 5: 1–2. 21472033 10.3389/fnint.2011.00002 PMC3068294

[18] Falter CM, Noreika V (2011) Interval timing deficits and abnormal cognitive development. Frontiers in Integrative Neuroscience 5: 1–2. 21716645 10.3389/fnint.2011.00026 PMC3116141

[19] Allman MJ, DeLeon IG, Wearden JH (2011) Psychophysical assessment of timing in individuals with autism. American Journal of Intellectual and Developmental Disabilities 116. 10.1352/1944-7558-116.2.165 PMC4822529 21381951

[20] Falter CM, Noreika V, Wearden JH, Bailey AJ (In Press) More consistent, yet less sensitive: interval timing in autism spectrum disorders. Quarterly Journal of Experimental Psychology. In press. 10.1080/17470218.2012.690770 22800511

[21] Gowen E, Miall C (2005) Behavioural aspects of cerebellar function in adults with Asperger syndrome. The Cerebellum 4: 1–11. 10.1080/14734220500355332 16321884

[22] Maister L, Plaisted-Grant KC (2011) Time perception and its relationship to memory in Autism Spectrum Conditions. Developmental Science 14: 1311–1322. 22010891 10.1111/j.1467-7687.2011.01077.x

[23] Martin JS, Poirier M, Bowler DM (2009) Brief report: Impaired temporal reproduction performance in adults with autism spectrum disorder. Journal of Autism and Developmental Disorders, DOI 10.10007/s10803-009-0904-3. 10.1007/s10803-009-0904-3 19924521

[24] Mostofsky SH, Goldberg MC, Landa RJ, Denckla MB (2000) Evidence for a deficit in procedural learning in children and adolescents with autism: implications for cerebellar contribution. Journal of the International Neuropsychological Society 6: 752–759. 11105465 10.1017/s1355617700677020

[25] Szelag E, Kowalska J, Galkowski T, Pöppel E (2004) Temporal processing deficits in high-functioning children with autism. British Journal of psychology 95: 269–282. 15296535 10.1348/0007126041528167

[26] Wallace GL, Happé F (2008) Time perception in autism spectrum disorders. Research in Autism Spectrum Disorders 2: 447–455.

[27] Gibbon J, Church RM, Meck W (1984) Scalar timing in memory. In: Gibbon J, Allan L, editors. Annals of the New Academy of Sciences, vol. 423: Timing and time perception. New York: New York Academy of Sciences. 52–77. 10.1111/j.1749-6632.1984.tb23417.x 6588812

[28] Gibbon J (1991) Origins of scalar timing. Learning and Motivation 22: 3–38.

[29] Cheng RK, Ali YM, Meck W (2007) Ketamine “unlocks” the reduced clock-speed effects of cocaine following extended training: Evidence for dopamine-glutamate interactions in timing and time perception. Neurobiology of Learning and Memory 88: 149–159. 17513138 10.1016/j.nlm.2007.04.005

[30] Droit-Volet S, Mermillod M, Cocenas-Silva R, Gil S (2010) The effect of expectancy of a threatening event on time perception in human adults. Emotion 10: 908–914. 21171761 10.1037/a0020258

[31] Droit-Volet S, Fayolle SL, Gil S (2011) Emotion and time perception: effect of film-induced mood. Frontiers in Intergrative Neuroscience doi: 10.3389/fnint.2011.00033. 10.3389/fnint.2011.00033 PMC3152725 21886610

[32] Gil S, Droit-Volet S (2012) Emotional time distortions: the fundamental role of arousal. Cognition and Emotion. doi:10.1080/02699931.2011.625401. 10.1080/02699931.2011.625401 22296278

[33] Maricq AV, Roberts S, Church RM (1981) Methamphetamine and time estimation. Journal of Experimental Psychology: Animal Behavior Processes 7: 18–30. 7229573 10.1037//0097-7403.7.1.18

[34] Lejeune H (1998) Switching or gating? The attentional challenge in cognitive models of psychological time. Behavioural Processes 44: 127–145. 24896971 10.1016/s0376-6357(98)00045-x

[35] Coull JT, Cheng RK, Meck WH (2011) Neuroanatomical and neurochemical substrates of timing. Neuropsychopharmacology 36: 3–25. 20668434 10.1038/npp.2010.113 PMC3055517

[36] Karmarkar UR, Buonomano DV (2007) Timing in the absence of clocks: Encoding time in neural network states. Neuron 53: 427–438. 17270738 10.1016/j.neuron.2007.01.006 PMC1857310

[37] Lewis PA, Miall RC (2003) Distinct systems for automatic and cognitively controlled time measurement: evidence from neuroimaging. Current Opinion in Neurobiology 13: 1–6. 10.1016/s0959-4388(03)00036-9 12744981

[38] Lewis PA, Miall RC (2006) A right hemispheric préfrontal system for cognitive time measurement. Behavioural Processes 71: 226–234. 16434151 10.1016/j.beproc.2005.12.009

[39] Macar F, Lejeune H, Bonnet M, Ferrara A, Pouthas V, et al (2002) Activation of the supplementary motor area and of attentional networks during temporal processing. Experimental Brain Research 142: 475–485. 11845243 10.1007/s00221-001-0953-0

[40] Gibbon J, Malapani C, Dale CL, Gallistel CR (1997) Toward a neurobiology of temporal cognition: advances and challenges. Current Opinion on Neurobiology 7: 170–184. 10.1016/s0959-4388(97)80005-0 9142762

[41] Lewis PA, Miall RC (2009) The precision of temporal judgement: milliseconds, many minutes, and beyond. Philosophical Transactions of the Royal Society 364: 1897–1905. 10.1098/rstb.2009.0020 PMC2685820 19487192

[42] Haldemann J, Stauffer C, Troche S, Rammsayer T (2012) Performance on auditory and visual temporal information processing is related to psychometric intelligence. Personality and Individual Differences 52: 9–14.

[43] Rammsayer TH, Brandler S (2007) Performance on temporal information processing as an index of general intelligence. Intelligence 35: 123–139.

[44] Garretson HB, Fein D, Waterhouse L (1990) Sustained attention in children with autism. Journal of Autism and Developmental Disorders 20: 101–114. 2324050 10.1007/BF02206860

[45] Vivanti G, Nadig A, Ozonoff S, Rogers SJ (2008) What do children with autism attend to during imitation tasks? Journal of Experimental Child Psychology 101: 186–205. 18582895 10.1016/j.jecp.2008.04.008 PMC6952170

[46] Droit-Volet S, Rattat AC (2007) A further analysis of time bisection behavior in children with and without reference memory: the similarity and the partition task. Acta Psychologica 125: 240–256. 17055990 10.1016/j.actpsy.2006.08.003

[47] Droit-Volet S, Wearden JH (2001) Temporal bisection in children. Journal of Experimental Child Psychology 80: 142–159. 11529672 10.1006/jecp.2001.2631

[48] Droit-Volet S, Wearden JH (2002) Speeding up an internal clock in children? Effects of visual flicker on subjective duration. Quarterly Journal of Experimental Psychology 55: 193–211. 12188524 10.1080/02724990143000252

[49] Rubia K, Smith A (2004) The neural correlates of cognitive time management: a review. Acta Neurobiologiae Experimentalis 64: 329–340. 15283476 10.55782/ane-2004-1517

[50] Allan LG, Gibbon J (1991) Human bisection at geometric mean. Learning and Motivation 22: 39–58.

[51] Gil S, Droit-Volet S (2011) “Time flies in the presence of angry faces”. … depending on the temporal task used! Acta Psychologica 136: 354–362. 21276583 10.1016/j.actpsy.2010.12.010

[52] Coull JT, Cheng RK, Meck WH (2011) Neuroanatomical and neurochemical substrates of timing. Neuropsychopharmacology 36: 3–25. 20668434 10.1038/npp.2010.113 PMC3055517

[53] Schopler E, Mesobov GB, Kunce LJ (Eds) (1998) Asperger’s Syndrome or High Functioning Autism? Plenum Press, ISBN 0–306–45746–6 New York.

[54] Simpson RL, Smith Myles B (Eds) (2008) Educating children and youth with autism: strategies for effective practice. (2nd edition) Pro Ed. ISBN -13: 978-1-4164-0210-7. Texas.

[55] Lejeune H, Wearden JH (2006) Scalar properties in animal timing: Conformity and violations. Quarterly Journal of Experimental Psychology 59: 1875–1908. 10.1080/17470210600784649 16987779

[56] Boucher J, Pons F, Lind S, William D (2007) Temporla cognition in childlren with autistic Spectrum disorders test of diachronic thinking. Journal of Autism and Developmental Disorders 37: 1413–1429. 17171540 10.1007/s10803-006-0285-9

[57] Hill E (2004) Executive dysfunction in autism. Trends in Cognitive Sciences 1: 26–32. 10.1016/j.tics.2003.11.003 14697400

[58] Gibbon J (1977) Scalar expectancy theory and Weber’s law in animal timing. Psychological Review 84: 279–325.

[59] Zélanti P, Droit-Volet S (2011) Cognitive abilities explaining age-related changes in time perception of short and long durations. Journal of Experimental Child Psychology 109: 143–157. 21334637 10.1016/j.jecp.2011.01.003

[60] Allan LG (2002) The location and interpretation of the bisection point. Quarterly Journal of Experimental Psychology 55B: 43–60. 10.1080/02724990143000162 11900306

[61] Baudouin A, Vanneste S, Isingrini M, Pouthas V (2006) Differential involvement of internal clock and working memory in the production and reproduction of duration: A study on older adults. Acta Psychologica 121: 285–296. 16139783 10.1016/j.actpsy.2005.07.004

[62] Corbett BA, Constantine LJ (2006) Autism and attention deficit hyperactivity : assessing attention and response control with the integrated visual and auditory continuous performance test. Child Neurpsychology 12: 335–348. 10.1080/09297040500350938 16911977

[63] Yoshida Y, Uchiyama T (2004) The clinical necessity for assessing attention deficit-hyperactivity disorder (ADHD) symptoms in children with high functioning Pervasive Developmental Disorder (PDD). European Child & Adolescent Psychiatry 13: 307–314. 15490278 10.1007/s00787-004-0391-1

[64] Droit-Volet S (In press). Time perception in children: A neurodevelopmental approach. Neuropsychologia. In press. 10.1016/j.neuropsychologia.2012.09.023 22999968

[65] Droit-Volet S, Zélanti P (2012) Time sensitivity in children and adults: duration ratios in bisection. Quarterly Journal of Experimental Psyhcology. 10.1080/17470218.2012.712148 22950870

[66] Zélanti P, Droit-Volet S (2012) Auditory and visual differences in time perception? An investigation from a developmental perspective with neuropsychological tests. Journal of Experimental Child Psychology. 10.1016/j.jecp.2012.01.003 22621934

[67] Courchesne E, Lincoln AJ, Yeung-Courchesne R, Elmasian R, Grillon C (1989) Pathophysiologic findings in nonretarded autism and receptive developmental language disorder. Journal of Autism and Developmental Disorders 19: 1–17. 2708293 10.1007/BF02212714

[68] Mann TA, Walker P (2003) Autism and a deficit in broadening the spread of visual attention. Journal of Child Psychology and Psychiatry 44: 274–284. 12587863 10.1111/1469-7610.00120

[69] Wearden JH, Edwards H, Fakhri M, Percival A (1998) Why sounds are judged longer than lights: Application of a model of the internal clock in humans. Quarterly Journal of Experimental Psychology 51B: 97–120. 10.1080/713932672 9621837

[70] Falter CM, Elliott MA, Bailey AJ (2012) Enhanced visual temporal resolution in autism spectrum disorders. PLoSONE 7: 1–6. 10.1371/journal.pone.0032774 PMC3309999 22470425

[71] Jones CRG, Happé F, Baird G, Simonoff E, Marsden AJS, et al (2009) Auditory discrimination and auditory sensory behaviours in autism spectrum disorders. Neuropsychologia 47: 2850–2858. 19545576 10.1016/j.neuropsychologia.2009.06.015

[72] Kwakye LD, Foss-Feig JH, Cascio CJ, Stone WL, Wallace MT (2011) Altered auditory and multisensory temporal processing in autism spectrum disorders. Frontiers in Integrative Neurosciences 4: 1–11. 10.3389/fnint.2010.00129 PMC3024004 21258617

[73] Hughes JR (2008) A review of recent reports on autism: 1000 studies published in 2007. Epilepsy & Behavior 12: 425–437. 10.1016/j.yebeh.2008.06.015 18627794

[74] Booth R, Happé F (2010) “Hunting with a knife and … fork”: Examining central coherence in autism, attention deficit/hyperactivity disorder, and typical development with a linguistic task. Journal of Experimental Child Psychology 107: 377–393. 20655060 10.1016/j.jecp.2010.06.003 PMC2941847

[75] Frith U (1989) Autism: Explaining the enigma. Oxford: Blackwell.

[76] Frith U, Happé F (1994) Autism: Beyond ‘theory of mind”. Cognition 50: 115–132. 8039356 10.1016/0010-0277(94)90024-8

[77] Glazebrook CM, Elliott D, Szatmari P (2008) How do individuals with autism plan their movements? Journal of Autism and Developmental Disorders 38: 114–126. 17436068 10.1007/s10803-007-0369-1

